# The effects of sitting Tai Chi on physical and psychosocial health outcomes among individuals with impaired physical mobility

**DOI:** 10.1097/MD.0000000000021805

**Published:** 2020-08-21

**Authors:** Jie Zhao, Janita Pak Chun Chau, Yuli Zang, Suzanne Hoi Shan Lo, Kai Chow Choi, Surui Liang

**Affiliations:** a The Nethersole School of Nursing, Faculty of Medicine, The Chinese University of Hong Kong, Shatin, New Territories, Hong Kong; b School of Nursing, Yunnan University of Traditional Chinese Medicine, Kunming, Yunnan, China.

**Keywords:** frail elderly, frailty, mobility limitation, musculoskeletal diseases, neurological rehabilitation, sitting Tai Chi, systematic review

## Abstract

**Background:**

Impaired physical mobility, most often seen in people with neurological disorders (i.e., stroke and spinal cord injury survivors), musculoskeletal diseases or frailty, is a limitation in independent and purposeful physical movement of the body or one or more extremities. The physical restrictions result in negative consequences on an individual's physical and psychosocial functions. This proposal describes a systematic review protocol to determine the effectiveness and approaches of sitting Tai Chi intervention for individuals with impaired physical mobility. Our review would inform stakeholders’ decisions in integrating this complementary therapy into current rehabilitation services.

**Methods:**

Randomized controlled trials or quasi-experimental studies that compared an intervention group receiving sitting Tai Chi with a control group among adult participants with impaired physical mobility resulting from any health condition(s) will be included. Outcomes of interest will include physical and psychosocial health outcomes. The Cochrane Central Register of Controlled Trials, MEDLINE, EMBASE, PubMed, CINAHL, Scopus, Web of Science, AMED, PsycINFO, SPORDiscus, PEDro, WanFang Data and China National Knowledge Infrastructure will be searched from their inception to January 2020. Additional searches will be performed to identify studies that are being refereed, to be published, unpublished or ongoing. Two reviewers will select the trials and extract data independently. The risk of bias of the included studies will be assessed using the Cochrane risk-of-bias tools. The Grading of Recommendations, Assessment, Development and Evaluation will be used to assess evidence quality for each review outcome. Data synthesis will be performed using Review Manager 5.3. When a meta-analysis is possible, we will assess the heterogeneity across the studies by computing the *I*^2^ statistics.

**Results:**

A high-quality synthesis of current evidence of sitting Tai Chi for impaired physical mobility will be stated from several aspect using subjective reports and objective measures of performance.

**Conclusion:**

This protocol will present the evidence of whether sitting Tai Chi is an effective intervention for impaired physical mobility.

**PROSPERO registration number:**

CRD 42019142681.

## 1 Introduction

### 1.1 Description of the condition – impaired physical mobility

Impaired physical mobility is defined as a limitation in independent and purposeful physical movement of the body or one or more extremities.^[1]^ It is characterized by alternations in gait; a decline in motor skills, range of motion and/or reaction time; difficulty in turning, engaging in substitutions for movement, postural instability, and slowed or uncoordinated movements.^[1]^ Physical mobility can be directly affected by disease processes including neurological disorders such as stroke and spinal cord injuries (SCI), and can be compounded by musculoskeletal and other conditions associated with ageing, such as frailty.^[2]^ Impaired physical mobility is strongly associated with activity intolerance, decrease in muscle control and muscle strength, and depressive symptoms.^[1]^

Impaired physical mobility has negative impacts on both physical and psychosocial functions. It may lead to deconditioning and loss of function of the whole body system, an increased risk of fall, body image disturbance, mood change, and limitations in activities of daily living (ADL) and social participation.^[2–4]^ Evidence suggests that impaired physical mobility and its associated complications may cause disability.^[2]^ Globally, the relative contribution of years lived with disability to the overall burden of disease was 33.5% of disability-adjusted life years in 2016.^[5]^ Disability is an important public health concern that is associated with greater risk of morbidity, hospitalization, mortality and increased healthcare expenditure.^[6]^ To prevent such a large range of negative outcomes, a population-focused holistic approach by targeting individuals with impaired physical mobility would be ideal.

### 1.2 Description of the intervention – Tai Chi and sitting Tai Chi

Holistic approaches have been developed to reduce disability and promote health among individuals with impaired physical mobility, including the use of complementary therapies such as Tai Chi (TC). TC is also known as Tai Ji or Tai Chi Chuan particularly among mainland Chinese, given its origin in ancient China as a type of martial arts underpinned by the Yin-Yang theory.^[7]^ TC runs as a series of repetitions of certain forms (for example, 12-form, 24-form and 48-form) of slow intentional movements. Desirable outcomes can be achieved if breathing and mental imagery are well synchronized with body movements, including but not limited to body and mind relaxation, personal growth and health improvement, and self-defense capability, mainly resulting from the enhanced flow of *qi* or inner energy according to the principles of traditional Chinese medicine.^[8]^

TC is one type of mind-body exercises that integrates physical, psychosocial, emotional, spiritual, and behavioral elements.^[7–9]^ Due to the multiple benefits, low cost, and easiness to learn without requirement of equipment for assistance, TC has grown into an appealing and popular physical activity of moderate intensity for rehabilitation or promotion of physical and psychosocial wellbeing, especially among those with chronic conditions.^[10]^ Recent evidence from systematic reviews further reveals the positive effects of TC on balance control and upper extremity function contributing to ADL execution, among older adults and stroke survivors.^[11,12]^ However, traditional TC requires a standing position and frequent body turning from one direction to the other. Individuals with impaired physical mobility resulting from stroke, brain/spinal injury or severe musculoskeletal trauma at the lower limbs face difficulties simultaneously maintaining a standing position and moving the torso and limbs. Consequently, traditional TC, while multi-beneficial, is infeasible. The decline in motor control skills and uncoordinated movements, common in these persons, increase the risk of falls.^[13,14]^ Evidence suggests that falls and fall-related injuries often result in a prolonged hospital stay and a substantially increased cost, which poses a significant burden on individuals, families, and healthcare systems.^[15,16]^ Perform TC from a sitting position may be a safer option to achieve similar benefits for those with impaired physical mobility. Sitting TC is a derivative or redesigned form of traditional TC practiced in a chair or a wheelchair, and first emerged in the 1990s.^[17,18]^

### 1.3 How the intervention (sitting Tai Chi) might work

Studies of energy expenditure indicate that sitting TC is an aerobic exercise.^[19]^ The US physical activity guidelines indicate that aerobic exercise can reduce the risk of functional limitations, including ADL dependency, and thus delay the onset of major disability.^[20]^ Kinematic evidence shows that sitting TC is a blend of slow helical movements that can tighten and strengthen extremities, the back and core muscles of the abdomen, and increase the flexibility of all joints involved, leading to enhancement of one's physical activity. If practiced in a group, sitting TC can facilitate social interactions among the group participants.^[19,21]^ An appropriate integration of deep breathing and mental concentration with body movements can generate a state of harmony between the body and the mind.^[22]^ In addition to the benefits to cardiovascular and pulmonary systems, the most frequently reported benefits of sitting TC are related to improving sitting balance, muscle strength, ADL, and psychosocial outcomes.^[23–25]^

### 1.4 Importance of this review

Regardless of underlying disease status, impaired physical mobility is often associated with disability and negative impact on individuals’ physical and psychosocial functions.^[2]^ Previous studies have examined the effects of sitting TC on physical and psychosocial health outcomes among individuals with impaired physical mobility. No review has been conducted to summarize the best available research evidence on the effects of sitting TC on health outcomes. There is also no consensus on the styles, forms, duration, frequency and practice strategies of sitting TC. It is imperative to examine thoroughly the intervention intensity and dosage of sitting TC for individuals with impaired physical mobility. The evidence will be used to guide future design, practice and evaluation of effective person-centered sitting TC programs.

## 2 Objectives

1. To systematically review the scientific literature and, if studies are sufficiently similar, perform a meta-analysis to determine the effects of sitting TC on physical and psychosocial health outcomes among individuals with impaired physical mobility.
2. To examine the factors influencing the success of the intervention including the styles and forms of sitting TC, the dose (intensity, duration, frequency), timing of delivery, delivery mode, and practice strategies of sitting TC.

## 3 Methods

The protocol has been registered in PROSPERO (CRD42019142681) and prepared according to the preferred reporting items for systematic reviews and meta-analysis protocols (PRISMA-P) checklist.^[26]^

### 3.1 Criteria for considering studies for this review

#### 3.1.1 Types of studies

All randomized controlled trials (RCTs) and quasi-experimental studies that compared an intervention group receiving TC in a sitting position with a control group will be included. There will be no restriction on the date of publication but the reporting language can only be English or Chinese. Non-experimental studies, qualitative studies, case studies, editorial comments, conference papers, literature reviews, systematic reviews, and abstracts will be excluded.

#### 3.1.2 Types of participants

The included will be adults of 18 years old or above who have impaired physical mobility caused by any health condition regardless of the severity of the underlying comorbidities. In this systematic review, impaired physical mobility is defined as a limitation in independent and purposeful physical movement of the body or one or more extremities.^[1]^ The underlying pathophysiolgoical problem may be associated with impaired muscle tone, muscular strength, or the limited range of motion of joints.

The related health conditions will include but not be limited to:

(1) Neurological disorders such as stroke and spinal cord injuries;
(2) Musculoskeletal diseases such as arthritis and gout; and
(3) Other conditions associated with ageing, such as frailty.

Those with particular health conditions, for instance vision or hearing diability, severe cognitive impairment, or traumatic injuries that may influence the practice of TC in a sitting position will be excluded.

#### 3.1.3 Types of interventions

We will include any form of physical activity intervention under the name of TC or its alternatives (e.g., Tai Ji, Tai Chi Chuan) performed in a sitting position. Such physical activity will have aimed to improve health and will have been practiced by individual participants alone or with others in a group format. The intervention could have been delivered in any setting through a single session or a series of sessions. Studies that included a co-intervention, for example, a systematically organized exercise program, will be excluded. As to the physical activity comparator, various counterpart physical activities for the control group will be considered, including null treatment, wait list controls, usual rehabilitation, or usual care.

#### 3.1.4 Types of outcome measures

No limitation will be set for the assessment methods, and the data collection could have occurred at any time points – before and/or after receiving the physical activity intervention. Primary and secondary outcomes identified for the proposed systematic review have been classified under three categories (Table 1).

**Table 1:**
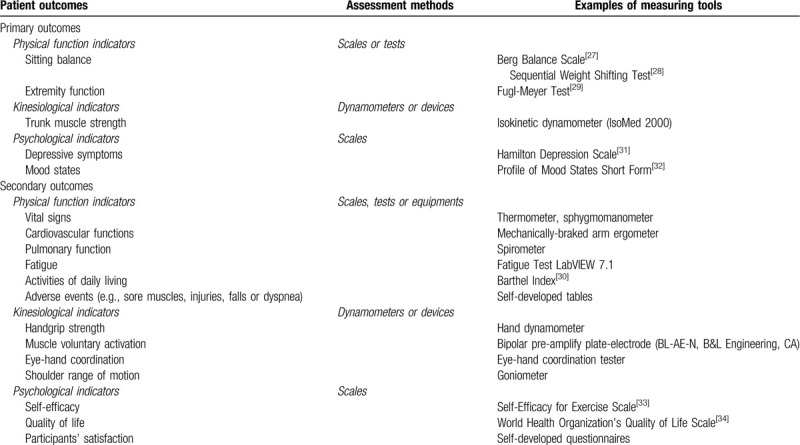
Proposed primary and secondary outcomes for the sitting Tai Chi intervention.

### 3.2 Search methods for identification of studies

We will search for relevant studies indexed in English or Chinese databases as listed below. Any newly emerging study meeting the aforementioned criteria during the entire period of this systematic review will be included. The MEDLINE strategy is presented in Table 2.

**Table 2:**
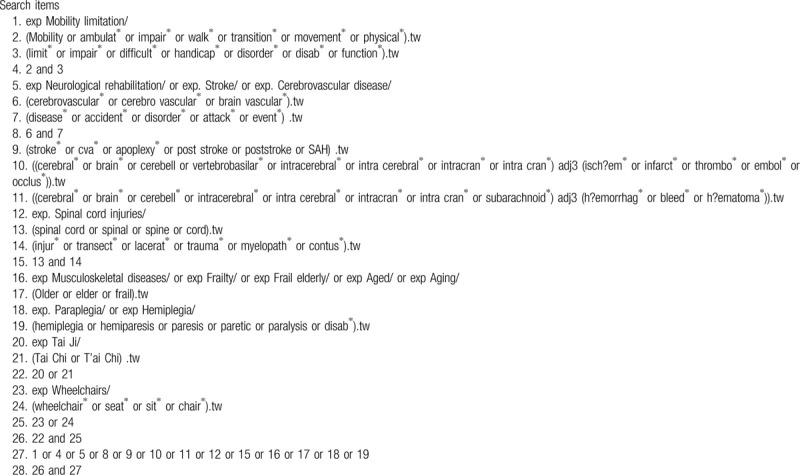
Ovid MEDLINE search strategy.

#### 3.2.1 Electronic searches

We will search the following 13 databases from their inception given the scarcity of evidence: The Cochrane Central Register of Controlled Trials (CENTRAL), MEDLINE (1946 to present), EMBASE (1910 to present), PubMed, CINAHL, Scopus (1823 to present), Web of Science, Allied and Complementary Medicine Database (AMED) (1985 to present), PsycINFO (1806 to present), SPORDiscus, PEDro, WanFang Data, and China National Knowledge Infrastructure (CNKI).

#### 3.2.2 Searching other resources

This search endeavor will aim to further identify published, unpublished and ongoing studies, in which we will:

(1) Search grey literature sources: Open Grey (www.opengrey.eu) and Grey Literature Report (www.greylit.org);
(2) Search trial registries: International Clinical Trials Registry Platform (ICTRP), World Health Organisation (WHO) (app.who.int/trialsearch), ClinicalTrials.gov, US National Institutes of Health (NIH) (http://clinicaltrials.gov/);
(3) Review the reference list of all included trials;
(4) Hand search relevant journals and conference proceedings;
(5) Contact experts and authors in this field who may have ongoing or unpublished research studies;
(6) Contact authors to obtain any missing or additional data; and
(7) Use the Scopus Citation Index for all included studies for tracking citations.

### 3.3 Data collection and analysis

#### 3.3.1 Selection of studies

**Figure 1:**
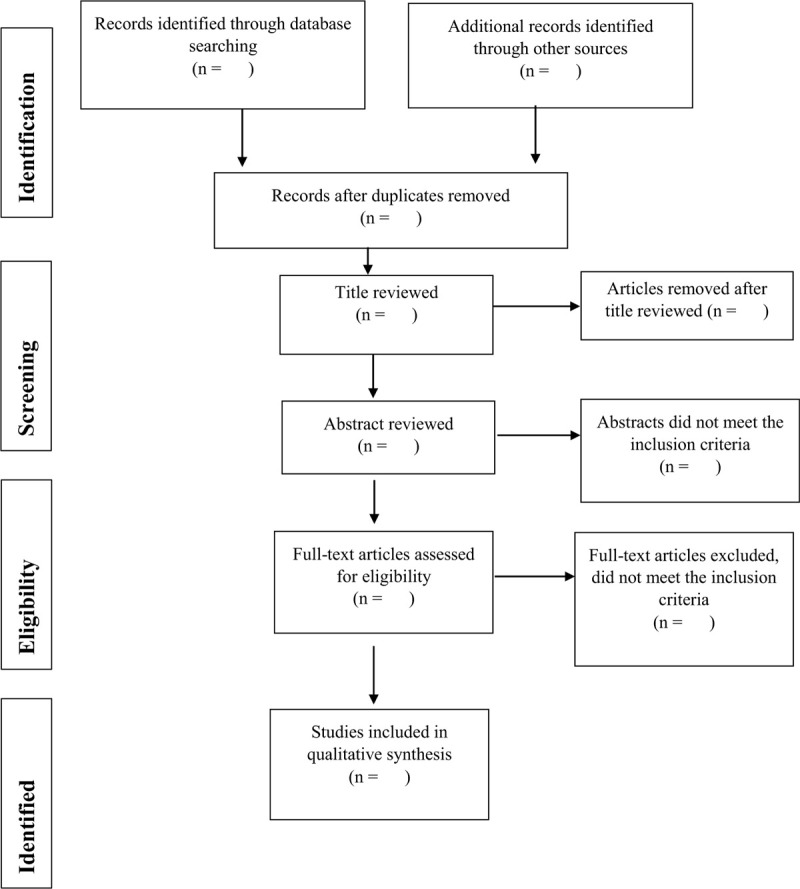
PRISMA flow chart of search process.^[46]^.

We will use the Cochrane Covidence to screen and extract data from the included trials.^[35]^ Two authors, JZ and SRL, will independently screen the eligible trials based on the titles and abstracts, and then examine the full text of potentially relevant articles to identify the most appropriate for inclusion using the proposed criteria. Disagreement between the two authors will be solved by consulting the co-authors (JPCC and SHSL). We will contact the original authors if more details are required for data pooling. We will collate multiple reports of the same study to avoid duplicated reporting. The PRISMA flowchart will be used to record the process of searching, screening and inclusion as well as the reason of exclusion (Fig. 1).

### 3.4 Data extraction and management

Two authors (JZ and SRL) will independently extract data from the included studies and document the following information using a pre-designed data collection template form containing the author, year of publication, country, setting, design, participants, interventions, comparators, outcome measures, and key findings. Before the independent extraction, three of the included studies will be randomly selected using a public online randomizer (http://www.randomisation.com/) to pilot-test the proposed extraction method to assure that the extracted items are clear enough and no important items are missed. We will approach the corresponding author for clarification of relevant details when necessary. Any disagreements regarding the extraction will be resolved through discussion with the co-authors (JPCC and SHSL), until a consensus is reached.

### 3.5 Assessment of risk of bias in included studies

Two authors, JZ and SRL, will independently evaluate the quality of the included trials through assessing the risk of bias using the following tools as appropriate:

(1) Version 2 of the Cochrane risk-of-bias tool (RoB 2) for randomized trials; and (2) the Risk of Bias in Non-randomized Studies of Interventions (ROBINS-I) tool for quasi-experimental studies with a control group.^[36,37]^
(2) Any disagreement will be solved using the same discussion and consensus approach as described in the previous sections.

The following aspects of risk bias for the included studies will be assessed: sequence generation, allocation concealment, blinding of participants, personnel and outcome assessors, incomplete outcome data, and selective outcome reporting. For RCTs, the three possible risk-of-bias judgment outcomes will be low risk of bias, some concerns, and high risk of bias.^[36]^ For quasi-experimental studies with a control group the possible risk-of-bias judgments will be low, moderate, serious, or critical risk of bias.^[37]^ We will illustrate the potential biases embedded in the included studies, individually and overall, using a ‘Risk of bias’ table, a graph, and a summary figure.

### 3.6 Measures of treatment effect

Outcome measures could be dichotomous or continuous. We will report the dichotomous variables (e.g., adverse events or no adverse events) as risk ratios (RRs) with 95% confidence intervals (CI). When measures are continuous, we will use mean differences (MDs) with 95% CI if the outcomes are measured using the same scale. Standardized mean differences (SMDs) with 95% CI will be adopted if different scales are used in different studies to measure the same outcome variable.

### 3.7 Unit of analysis issues

We will consider the analysis of outcomes based on the individual as the unit of analysis in this review. For studies with a cluster randomized design, we will include the study, if appropriate data analysis was performed, to extract effect estimates and their standard errors for meta-analysis. If not, we will perform appropriate analyses of cluster RCTs by reducing the size of each study to its effective sample size. The effective sample size will be adjusted by using the mean cluster size and intracluster correlation coefficient (ICC).^[38]^ In addition, we will classify studies with repeated measures as intermediate-, short,-or long-term if the measurement was performed immediately, two weeks to six months, or more than 6 months after the intervention, respectively.^[39]^ The meta-analysis will be performed using the intermediate-, short-, or long-term time frame as the classification variable.

### 3.8 Dealing with missing data

We will contact the original authors of included studies to request for missing data. If the missing data are not available, the studies will be excluded from further analysis and the potential impact of missing data will be addressed in the discussion section of this review.

### 3.9 Assessment of heterogeneity

We will assess the heterogeneity between studies through the visual inspection of forest plots, a chi-square test with a significance level at *P* < .10, and the *I*^*2*^ statistic. A threshold of *I*^*2*^ greater than 50% will be adopted to indicate the statistical heterogeneity.^[40]^ We will assess the methodological heterogeneity including differences in study design and the studies’ risk of bias. Participants’ characteristics, interventions, outcomes, and the settings in the included studies will be assessed to evaluate the extent of clinical heterogeneity.

### 3.10 Assessment of reporting biases

We will try to avoid reporting biases using a comprehensive search strategy to identify any eligible published studies, grey literature, and registers of prospective trials.^[41]^ When appropriate, we will detect reporting biases by the visual inspection of funnel plots.

### 3.11 Data synthesis

If more than two controlled trials are included, we will perform a meta-analysis under the condition that the included studies are sufficiently similar. We will use the generic inverse-variance and Mantel-Haenszel methods to combine continuous and dichotomous data for the meta-analysis respectively. A random-effect model will be used for meta-analysis if *I*^*2*^ *>* 50%; elsewhere, a fixed-effect model will be adopted. Other study characteristics and results will be summarized narratively if the meta-analysis cannot be performed for all or some of the included studies.

One author (JZ) will enter the data into Review Manager 5.3, and SRL will check the accuracy of the entries.^[42]^ For any emerging inconsistency or disagreement, the same discussion and consensus approach as described earlier will be undertaken.

### 3.12 Sensitivity analysis

Sensitivity analysis will be performed, if applicable, by comparing the results of meta-analyses that include or exclude the dubious studies to test the robustness of the results.^[40]^ The dubious studies will be those that meet at least one of the following criteria:

(1) no reporting of or inadequate/unclear approaches to methodology quality (sequence generation, allocation concealment, or blinding of participants or assessors);
(2) dubious sample size (small sample size trials, e.g., below 40 in each group) ^[43]^; and
(3) no reporting of or inadequate/unclear approaches to dealing with missing data.

### 3.13 Subgroup analysis

If there is a sufficient number of included studies, subgroup analysis will be performed by age (younger adults of < 44 years, middle-aged adults of 45 to 64 years, and older adults of > 65 years), type of impaired physical mobility associated with different health conditions, style/dose/form of sitting TC (e.g., 8 forms, 10 forms, 12 forms), and control group intervention (e.g., usual rehabilitation or usual care).

### 3.14 Confidence in cumulative evidence

The quality of the evidence will be assessed using the Grades of Recommendation, Assessment, Development and Evaluation (GRADE) evidence grading system in the aspects of methodological quality, directness of evidence, heterogeneity, precision of effect estimates, and risk of publication bias.^[44]^

## 4 Discussion

Impaired physical mobility can lead to the deconditioning and impairment of both physical and psychosocial functions.^[2–4]^ Sitting TC has been claimed to have many potential benefits, including the reduction of disability and the promotion of physical and mental health among individuals with impaired physical mobility.^[23–25]^ It is suitable for implementation as an early rehabilitation intervention to enable individuals to improve or maintain their physical and psychosocial functioning. Sitting TC can be performed as a community- or home-based health promotion action. This is particularly important for developing countries, where there is a prevalent shortage of resources for hospital- and community-based rehabilitation.^[45]^

While sitting TC does not involve the movement of lower limbs, people with impaired physical mobility may still find maintaining balance during upper body movements and concurrently harmonizing mental and physical efforts challenging. A clear understanding of the styles, forms, and doses of sitting TC is necessary to establish practice guidelines for the rehabilitation of individuals with impaired physical mobility and facilitate the prescription of sitting TC as an exercise management regime for them.

However, there is a lack of cumulative evidence on the effectiveness and the practice of sitting TC on the physical and psychosocial health outcomes among individuals with impaired physical mobility. Without strong evidence, the use of this potentially beneficial intervention will not be taken into consideration by health professionals or possible beneficiaries. To the best of our knowledge, this will be the first systematic review to summarize the existing evidence on the use of sitting TC for health among individuals with impaired physical mobility. It is anticipated that the adoption of a transparent and rigorous systematic review will result in the assembly of the best evidence for the application of sitting TC among the target population. In addition, reporting the limitations of the included studies will guide future efforts to generate more robust research outcomes. The publication of convincing evidence on the effectiveness of sitting TC and the best way to practice it will raise the public's and health professionals’ awareness, leading to a wider application among the target population.

The greatest potential stumbling block to this review might be the scarcity of existing studies meeting the inclusion criteria. We have planned comprehensive search strategies, as described above, to search 13 databases to overcome this risk. Another threat to the synthesis of evidence might be the wide variation in the underlying disease status, increasing the heterogeneity. A narrative description of the identified findings will be a potential alternative solution to the challenge of meta-analysis.

In conclusion, our review will provide useful evidence for researchers, health professionals, policy-makers and potential users of sitting TC to make effective decisions to integrate this physical activity for rehabilitation and health promotion.

## Author contributions

JZ is the guarantor. JZ, JPCC and YLZ, wrote the paper, while SHSL and KCC critically reviewed and revised the paper. JZ and SRL will conduct the search, data screening, and data extraction with JPCC and YLZ's close supervision and support. All authors approved the final version of the manuscript.
